# 2-(4-Oxo-3-phenyl-1,3-thia­zolidin-2-yl­idene)malononitrile

**DOI:** 10.1107/S160053681300216X

**Published:** 2013-02-06

**Authors:** Ola K. Sakka, Daisy H. Fleita, William T. A. Harrison

**Affiliations:** a Department of Chemistry, American University in Cairo, PO Box 74, New Cairo 11835, Egypt; b Department of Chemistry, University of Aberdeen, Meston Walk, Aberdeen AB24 3UE, Scotland

## Abstract

In the title compound, C_12_H_7_N_3_OS, the essentially planar thia­zole ring (r.m.s. deviation = 0.022 Å) forms dihedral angles of 84.88 (9) and 1.8 (3)° with the phenyl ring and the –C(CN)_2_ group (r.m.s. deviation = 0.003 Å), respectively. The mol­ecule has approximate local *C*_s_ symmetry. In the crystal, molecules are linked *via* C—H⋯N hydrogen bonds, forming chains propagating along [101]. The crystal studied was found to be an inversion twin with a refined 0.63 (1):0.37 (1) domain ratio.

## Related literature

For background to 1,3-thia­zolidin-4-ones and their properties, see: Singh *et al.* (1981 ▶); Liesen *et al.* (2010 ▶); Kocabalkanli *et al.* (2001 ▶); Kumar *et al.* (2007 ▶). For further synthetic details, see: Mohareb *et al.* (2012 ▶). For a related structure, see: Pomés Hernández *et al.* (1996 ▶).
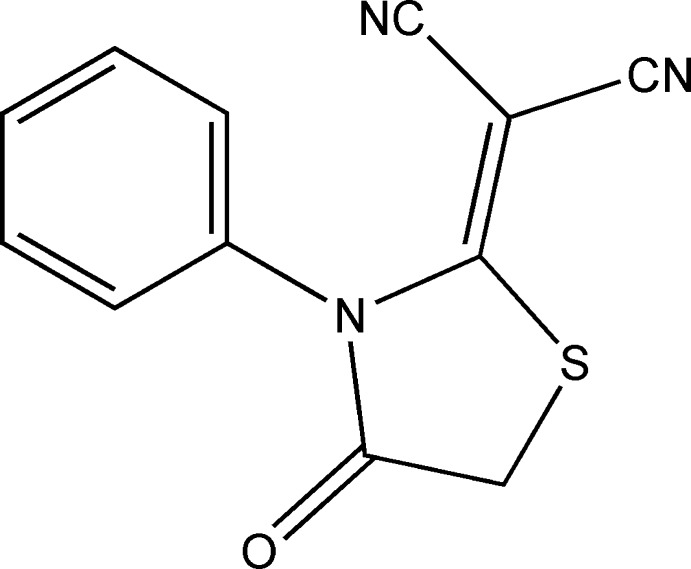


## Experimental

### 

#### Crystal data

C_12_H_7_N_3_OS
*M* *_r_* = 241.27
Monoclinic, 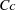

*a* = 17.0305 (8) Å
*b* = 9.5638 (6) Å
*c* = 7.1651 (4) Å
β = 104.199 (4)°
*V* = 1131.37 (11) Å^3^
*Z* = 4
Mo *K*α radiation
μ = 0.27 mm^−1^
*T* = 298 K
0.20 × 0.13 × 0.05 mm

#### Data collection

Nonius KappaCCD diffractometer
2136 measured reflections
2136 independent reflections
1397 reflections with *I* > 2σ(*I*)
*R*_int_ = 0.029

#### Refinement

*R*[*F* ^2^ > 2σ(*F* ^2^)] = 0.043
*wR*(*F* ^2^) = 0.086
*S* = 1.01
2136 reflections
156 parameters
2 restraints
H-atom parameters constrained
Δρ_max_ = 0.17 e Å^−3^
Δρ_min_ = −0.18 e Å^−3^
Absolute structure: Flack (1983 ▶), 835 Friedel pairs
Flack parameter: 0.37 (1)

### 

Data collection: *COLLECT* (Nonius, 1998 ▶); cell refinement: *SCALEPACK* (Otwinowski & Minor, 1997 ▶); data reduction: *DENZO* (Otwinowski & Minor, 1997 ▶), *SCALEPACK* and *SORTAV* (Blessing, 1995 ▶); program(s) used to solve structure: *SHELXS97* (Sheldrick, 2008 ▶); program(s) used to refine structure: *SHELXL97* (Sheldrick, 2008 ▶); molecular graphics: *ORTEP-3* (Farrugia, 2012 ▶); software used to prepare material for publication: *SHELXL97*.

## Supplementary Material

Crystal structure: contains datablock(s) global, I. DOI: 10.1107/S160053681300216X/lh5577sup1.cif

Structure factors: contains datablock(s) I. DOI: 10.1107/S160053681300216X/lh5577Isup2.hkl

Supplementary material file. DOI: 10.1107/S160053681300216X/lh5577Isup3.cml

Additional supplementary materials:  crystallographic information; 3D view; checkCIF report

## Figures and Tables

**Table 1:** Hydrogen-bond geometry (Å, °)

*D*—H⋯*A*	*D*—H	H⋯*A*	*D*⋯*A*	*D*—H⋯*A*
C10—H10⋯N2^i^	0.93	2.62	3.504 (5)	159

Symmetry code: (i) 
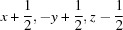
.

**Fig. 1.**
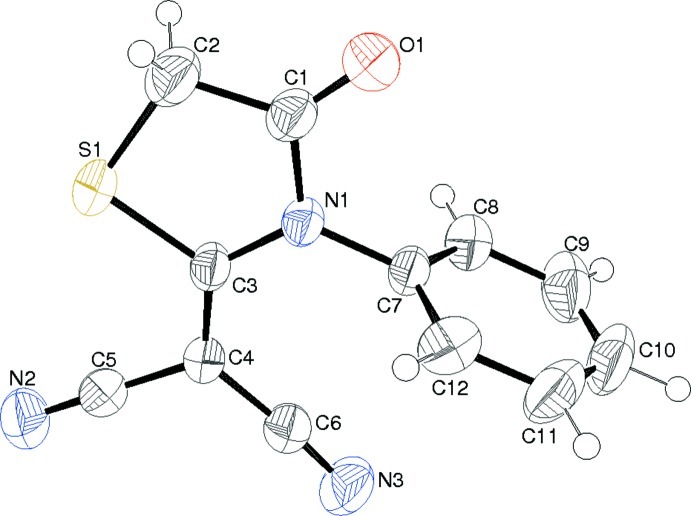
The molecular structure of the title compound, showing displacement ellipsoids at the 50% probability level.

## supplementary crystallographic information

### Comment

1,3-Thiazolidin-4-ones are heterocyclic compounds that have an atom of sulfur and nitrogen at positions 1 and 3, respectively and a carbonyl group at position 4. Their presence in penicillin was the first recognition of their occurrence in nature (Singh *et al.*, 1981). They have found uses as antibacterial (Liesen *et al.*, 2010), antimicrobial (Kocabalkanli *et al.*, 2001) and anti-inflammatory agents (Kumar *et al.*, 2007). In continuation of our studies on this family of compounds, we now report the synthesis and crystal structure of the title compound, (I).

The heterocyclic ring (C1/C2/S1/C3/N1) in (I) is close to planar (r.m.s. deviation = 0.022 Å) and subtends dihedral angles with the phenyl ring and the C(CN)_2_ group (r.m.s. deviation = 0.003 Å) of 84.88 (9) and 1.8 (3)°, respectively. An alternative analysis of the heterocyclic ring as a shallow envelope sees the S atom in the flap position displaced by 0.090 (5) Å from the other atoms (r.m.s. deviation = 0.004 Å). The molecule has approximate local C_s_ symmetry.

There is a short intermolecular contact [2.903 (2) Å] from the O atom of the carbonyl group to the centre of a nearby heterocyclic ring (symmetry: *x*, 1 - *x*, *z* - 1/2), but given the non-aromatic nature of the ring, this can hardly be regarded as a bond. No other significant intermolecular contacts occur in the crystal.

For a related structure, in which the thiazole ring was also found to be almost planar, see Pomés Hernández *et al.* (1996).

### Experimental

To a solution of malononitrile (0.66 g, 0.01 mol) dissolved in dimethylformamide (15 ml), potassium hydroxide pellets (0.56 g, 0.01 mol) and phenylisothiocyanate (1.35 g, 0.01 mol) were added. The reaction mixture was covered and stirred at room temperature overnight. *N*'-(2-chloroacetyl)-2-cyanoacetohydrazide (1.75 g, 0.01 mol) (Mohareb *et al.*, 2012) was stirred in the following day, and the solution was covered for another night, after which the reaction mixture was poured onto ice, neutralized with dil. HCl and the precipitated solid filtered off. Yellow blocks were obtained by slow evaporation of an ethanol solution.

### Refinement

The H atoms were placed in calculated positions (C—H = 0.93–0.97 Å) and refined as riding. The constraint *U*_iso_(H) = 1.2*U*_eq_(C) was applied.

### Crystal data

**Table:**

C_12_H_7_N_3_OS	*F*(000) = 496
*M**_r_* = 241.27	*D*_x_ = 1.416 Mg m^−^^3^
Monoclinic, *C**c*	Mo *K*α radiation, λ = 0.71073 Å
Hall symbol: C -2yc	Cell parameters from 1133 reflections
*a* = 17.0305 (8) Å	θ = 2.9–27.5°
*b* = 9.5638 (6) Å	µ = 0.27 mm^−^^1^
*c* = 7.1651 (4) Å	*T* = 298 K
β = 104.199 (4)°	Block, yellow
*V* = 1131.37 (11) Å^3^	0.20 × 0.13 × 0.05 mm
*Z* = 4	

### Data collection

**Table:**

Nonius KappaCCD diffractometer	1397 reflections with *I* > 2σ(*I*)
Radiation source: fine-focus sealed tube	*R*_int_ = 0.029
Graphite monochromator	θ_max_ = 27.5°, θ_min_ = 3.6°
ω scans	*h* = −21→22
2136 measured reflections	*k* = −12→11
2136 independent reflections	*l* = −9→9

### Refinement

**Table:**

Refinement on *F*^2^	Hydrogen site location: inferred from neighbouring sites
Least-squares matrix: full	H-atom parameters constrained
*R*[*F*^2^ > 2σ(*F*^2^)] = 0.043	*w* = 1/[σ^2^(*F*_o_^2^) + (0.0223*P*)^2^ + 0.0674*P*] where *P* = (*F*_o_^2^ + 2*F*_c_^2^)/3
*wR*(*F*^2^) = 0.086	(Δ/σ)_max_ < 0.001
*S* = 1.01	Δρ_max_ = 0.17 e Å^−^^3^
2136 reflections	Δρ_min_ = −0.18 e Å^−^^3^
156 parameters	Extinction correction: *SHELXL97* (Sheldrick, 2008), Fc^*^=kFc[1+0.001xFc^2^λ^3^/sin(2θ)]^-1/4^
2 restraints	Extinction coefficient: 0.008 (2)
Primary atom site location: structure-invariant direct methods	Absolute structure: Flack (1983), 835 Friedel pairs
Secondary atom site location: difference Fourier map	Flack parameter: 0.37 (1)

### Special details

**Table:**

Geometry. All e.s.d.'s (except the e.s.d. in the dihedral angle between two l.s. planes) are estimated using the full covariance matrix. The cell e.s.d.'s are taken into account individually in the estimation of e.s.d.'s in distances, angles and torsion angles; correlations between e.s.d.'s in cell parameters are only used when they are defined by crystal symmetry. An approximate (isotropic) treatment of cell e.s.d.'s is used for estimating e.s.d.'s involving l.s. planes.
Refinement. Refinement of *F*^2^ against ALL reflections. The weighted *R*-factor *wR* and goodness of fit *S* are based on *F*^2^, conventional *R*-factors *R* are based on *F*, with *F* set to zero for negative *F*^2^. The threshold expression of *F*^2^ > σ(*F*^2^) is used only for calculating *R*-factors(gt) *etc*. and is not relevant to the choice of reflections for refinement. *R*-factors based on *F*^2^ are statistically about twice as large as those based on *F*, and *R*- factors based on ALL data will be even larger.

### Fractional atomic coordinates and isotropic or equivalent isotropic displacement parameters (Å^2^)

**Table:**

	*x*	*y*	*z*	*U*_iso_*/*U*_eq_	
C1	0.40086 (17)	0.3723 (3)	0.3314 (5)	0.0435 (8)	
C2	0.31911 (18)	0.3591 (4)	0.3709 (4)	0.0542 (9)	
H2A	0.2827	0.3104	0.2657	0.065*	
H2B	0.2969	0.4511	0.3830	0.065*	
C3	0.43425 (18)	0.2567 (3)	0.6297 (4)	0.0357 (7)	
C4	0.48151 (17)	0.1984 (3)	0.7933 (4)	0.0395 (8)	
C5	0.4414 (2)	0.1387 (4)	0.9303 (5)	0.0487 (8)	
C6	0.5676 (2)	0.1857 (3)	0.8434 (5)	0.0444 (8)	
C7	0.54465 (17)	0.3210 (3)	0.4754 (4)	0.0368 (7)	
C8	0.58860 (18)	0.4396 (4)	0.5395 (4)	0.0465 (8)	
H8	0.5650	0.5143	0.5884	0.056*	
C9	0.6685 (2)	0.4455 (5)	0.5297 (5)	0.0669 (11)	
H9	0.6994	0.5242	0.5746	0.080*	
C10	0.7026 (2)	0.3359 (5)	0.4543 (5)	0.0689 (13)	
H10	0.7563	0.3411	0.4470	0.083*	
C11	0.6581 (2)	0.2197 (5)	0.3901 (5)	0.0657 (12)	
H11	0.6816	0.1456	0.3394	0.079*	
C12	0.5784 (2)	0.2111 (4)	0.3998 (4)	0.0534 (10)	
H12	0.5480	0.1318	0.3557	0.064*	
S1	0.32943 (5)	0.26310 (9)	0.59038 (11)	0.0515 (3)	
O1	0.41623 (12)	0.4253 (2)	0.1924 (3)	0.0585 (7)	
N1	0.46104 (13)	0.3139 (3)	0.4828 (3)	0.0363 (6)	
N2	0.40992 (18)	0.0906 (3)	1.0384 (5)	0.0703 (9)	
N3	0.63584 (18)	0.1710 (3)	0.8969 (4)	0.0671 (9)	

### Atomic displacement parameters (Å^2^)

**Table:**

	*U*^11^	*U*^22^	*U*^33^	*U*^12^	*U*^13^	*U*^23^
C1	0.0331 (18)	0.039 (2)	0.0570 (19)	−0.0008 (15)	0.0092 (15)	−0.0014 (17)
C2	0.036 (2)	0.071 (3)	0.0535 (19)	0.0005 (17)	0.0065 (14)	0.0031 (18)
C3	0.0287 (16)	0.0350 (19)	0.0452 (17)	−0.0036 (15)	0.0126 (13)	−0.0012 (14)
C4	0.0327 (18)	0.042 (2)	0.0459 (19)	0.0010 (14)	0.0142 (15)	0.0074 (15)
C5	0.0362 (19)	0.050 (2)	0.0577 (19)	0.0011 (16)	0.0074 (16)	0.0099 (17)
C6	0.040 (2)	0.048 (2)	0.0461 (17)	−0.0002 (16)	0.0109 (14)	0.0044 (17)
C7	0.0283 (17)	0.045 (2)	0.0386 (17)	−0.0005 (15)	0.0110 (13)	0.0027 (15)
C8	0.042 (2)	0.051 (2)	0.0490 (18)	−0.0055 (17)	0.0144 (14)	−0.0015 (16)
C9	0.047 (2)	0.095 (3)	0.060 (2)	−0.020 (2)	0.0139 (18)	0.007 (2)
C10	0.030 (2)	0.122 (4)	0.057 (2)	0.003 (2)	0.0147 (16)	0.012 (2)
C11	0.046 (2)	0.098 (3)	0.057 (2)	0.026 (2)	0.0199 (19)	0.003 (2)
C12	0.049 (2)	0.057 (3)	0.052 (2)	0.0062 (18)	0.0080 (17)	−0.0045 (15)
S1	0.0288 (4)	0.0603 (6)	0.0661 (5)	−0.0030 (5)	0.0131 (3)	0.0097 (5)
O1	0.0528 (15)	0.0679 (18)	0.0571 (13)	0.0059 (12)	0.0178 (10)	0.0173 (13)
N1	0.0280 (14)	0.0374 (16)	0.0442 (14)	−0.0027 (11)	0.0103 (11)	0.0005 (12)
N2	0.0528 (19)	0.085 (3)	0.076 (2)	−0.0039 (18)	0.0208 (15)	0.030 (2)
N3	0.040 (2)	0.092 (3)	0.067 (2)	0.0083 (17)	0.0091 (15)	0.0192 (18)

### Geometric parameters (Å, º)

**Table:**

C1—O1	1.202 (3)	C7—C12	1.372 (4)
C1—N1	1.412 (4)	C7—C8	1.374 (4)
C1—C2	1.492 (4)	C7—N1	1.439 (3)
C2—S1	1.792 (3)	C8—C9	1.381 (4)
C2—H2A	0.9700	C8—H8	0.9300
C2—H2B	0.9700	C9—C10	1.372 (6)
C3—N1	1.361 (4)	C9—H9	0.9300
C3—C4	1.367 (4)	C10—C11	1.361 (5)
C3—S1	1.739 (3)	C10—H10	0.9300
C4—C6	1.426 (4)	C11—C12	1.378 (5)
C4—C5	1.444 (5)	C11—H11	0.9300
C5—N2	1.142 (4)	C12—H12	0.9300
C6—N3	1.140 (4)		
			
O1—C1—N1	122.6 (3)	C7—C8—C9	118.7 (3)
O1—C1—C2	126.5 (3)	C7—C8—H8	120.7
N1—C1—C2	110.9 (3)	C9—C8—H8	120.7
C1—C2—S1	108.3 (2)	C10—C9—C8	120.5 (4)
C1—C2—H2A	110.0	C10—C9—H9	119.8
S1—C2—H2A	110.0	C8—C9—H9	119.8
C1—C2—H2B	110.0	C11—C10—C9	120.1 (4)
S1—C2—H2B	110.0	C11—C10—H10	119.9
H2A—C2—H2B	108.4	C9—C10—H10	119.9
N1—C3—C4	126.1 (3)	C10—C11—C12	120.4 (4)
N1—C3—S1	112.7 (2)	C10—C11—H11	119.8
C4—C3—S1	121.2 (2)	C12—C11—H11	119.8
C3—C4—C6	127.1 (2)	C7—C12—C11	119.2 (4)
C3—C4—C5	117.8 (3)	C7—C12—H12	120.4
C6—C4—C5	115.1 (3)	C11—C12—H12	120.4
N2—C5—C4	179.5 (4)	C3—S1—C2	92.06 (15)
N3—C6—C4	174.6 (3)	C3—N1—C1	115.8 (2)
C12—C7—C8	121.1 (3)	C3—N1—C7	124.7 (2)
C12—C7—N1	119.5 (3)	C1—N1—C7	119.4 (2)
C8—C7—N1	119.3 (3)		

### Hydrogen-bond geometry (Å, º)

**Table:**

*D*—H···*A*	*D*—H	H···*A*	*D*···*A*	*D*—H···*A*
C10—H10···N2^i^	0.93	2.62	3.504 (5)	159

Symmetry code: (i) *x*+1/2, −*y*+1/2, *z*−1/2.
